# Asymmetrical Plasmon Distribution in Hybrid AuAg Hollow/Solid Coded Nanotubes

**DOI:** 10.3390/nano13060992

**Published:** 2023-03-09

**Authors:** Aziz Genç, Javier Patarroyo, Jordi Sancho-Parramon, Raul Arenal, Neus G. Bastús, Victor Puntes, Jordi Arbiol

**Affiliations:** 1 Catalan Institute of Nanoscience and Nanotechnology (ICN2), CSIC and BIST, Campus Universitat Autònoma de Barcelona, 08193 Barcelona, Spain; 2 Rudjer Boskovic Institute, 10000 Zagreb, Croatia; 3 Instituto de Nanociencia y Materiales de Aragon (INMA), CSIC-U de Zaragoza, 50009 Zaragoza, Spain; 4 Laboratorio de Microscopias Avanzadas (LMA), Universidad de Zaragoza, 50018 Zaragoza, Spain; 5 ARAID Foundation, 50018 Zaragoza, Spain; 6 Vall d’Hebron Institut de Recerca (VHIR), 08035 Barcelona, Spain; 7 ICREA, 08010 Barcelona, Spain

**Keywords:** plasmon coded, nanotubes, nanowires, asymmetrical distribution, metal nanotubes, electron energy-loss spectroscopy, AuAg, localized surface plasmon resonances, boundary element method

## Abstract

Morphological control at the nanoscale paves the way to fabricate nanostructures with desired plasmonic properties. In this study, we discuss the nanoengineering of plasmon resonances in 1D hollow nanostructures of two different AuAg nanotubes, including completely hollow nanotubes and hybrid nanotubes with solid Ag and hollow AuAg segments. Spatially resolved plasmon mapping by electron energy loss spectroscopy (EELS) revealed the presence of high order resonator-like modes and localized surface plasmon resonance (LSPR) modes in both nanotubes. The experimental findings accurately correlated with the boundary element method (BEM) simulations. Both experiments and simulations revealed that the plasmon resonances are intensely present inside the nanotubes due to plasmon hybridization. Based on the experimental and simulated results, we show that the novel hybrid AuAg nanotubes possess two significant coexisting features: (i) LSPRs are distinctively generated from the hollow and solid parts of the hybrid AuAg nanotube, which creates a way to control a broad range of plasmon resonances with one single nanostructure, and (ii) the periodicity of the high-order modes are disrupted due to the plasmon hybridization by the interaction of solid and hollow parts, resulting in an asymmetrical plasmon distribution in 1D nanostructures. The asymmetry could be modulated/engineered to control the coded plasmonic nanotubes.

## 1. Introduction

Surface plasmon resonances are collective oscillations of conduction electrons in a material excited by an electromagnetic wave [1]. Such a unique property has enabled the usage of plasmonic nanostructures as building blocks for nanooptics and various novel applications, including, but not limited to, sensor devices [2,3], surface-enhanced Raman spectroscopy (SERS) [4,5], biomedicine [6], and photovoltaics [7,8], due to their ability to localize light at the nanoscale that is far beyond the diffraction limit of visible electromagnetic waves in dielectric media [9,10].

Localized surface plasmon resonances can direct and enhance radiative emission (and vice versa) in transmission mode, and they can convert the propagating freespace EM wave to highly confined and strongly enhanced electric fields [11,12,13,14,15,16]. Plasmonic nanostructures can be defined as nanoantennas as their EM modulation mechanism is similar to the radio antennas [11,12]. Gold and silver nanostructures are promising nanoantennas with good metallic properties [14,17]. Due to their ability to convert light into highly localized fields, plasmonic nanoantennas have been used to enhance the performance of various photoactive devices, such as solar cells, photodetectors, and biosensors [7,18,19]. They are also widely used in nanophotonic circuits as they can modify the amount and direction of emitted electromagnetic energy [20,21,22].

Electron energy-loss spectroscopy (EELS) has been widely used to obtain the plasmonic and electronic properties of various nanostructures [23,24,25]. Among the different metal nanostructures analyzed via EELS, the plasmonic properties of Ag nanorods/nanowires are intensively studied [26,27,28,29,30,31]. However, there is little information about the plasmonic properties of 1D hollow metal nanostructures in the literature. In general, hollow metal nanostructures exhibit enhanced plasmonic properties [32,33,34,35] due to a mechanism called plasmon hybridization between the solid parts and voids of the nanostructures [36]. S. Yazdi et al. [37] reported the controllable, reversible, and dynamic tuning of plasmon resonances in partially hollow AgAu nanorods via EELS, which showed the extent of the ability to control plasmonic properties in hollow nanostructures [37]. The idea of having repeated units, i.e., coded nanostructures, to have better properties is also applicable to other optical devices [38]. To further investigate this phenomenon by comparing the plasmonic properties of hollow and partially hollow nanostructures, the present study reports the observations of plasmon resonances in 1D hollow nanostructures of two different AuAg nanotubes. These nanotubes include completely hollow nanotubes and hybrid nanotubes comprising the sequential formation of solid Ag parts and hollow AuAg parts. These novel AuAg nanotubes can be good alternatives to the above-mentioned applications as they possess controllable and enhanced plasmonic properties that cover ultraviolet, visible, and near-infrared ranges in a single nanostructure.

## 2. Methods

The present study reports the plasmonic properties and asymmetrical plasmon distributions in the 1D hollow nanostructures of two different AuAg nanotubes, including completely hollow nanotubes and hybrid solid/hollow coded nanotubes comprising the sequential formation of solid Ag parts and hollow AuAg parts. In this section, we present the study’s methods, such as the synthesis of these nanostructures and the characterization of their plasmonic properties by STEM-EELS analyses and corresponding boundary element method (BEM) simulation studies.

### 2.1. Synthesis of 1D AuAg Nanostructures

The syntheses of AuAg nanotubes were produced by the galvanic replacement reaction of Ag nanowires used as templates following a similar methodology previously described in [39]. In the present case, several micron-long, penta-twinned Ag nanowires with a diameter of about 80 nm were synthesized via a solution-phase approach, as reported by Sun et al. [40], and used as templates. In a typical synthesis of completely hollow AuAg nanotubes, 0.25 mL of Ag nanowires (310 ppm Ag^+^, 2.9 mM by ICP-MS) were dispersed in a solution containing 2 mL of milli-Q water, 1 mL of CTAB (14 mM), and 0.1 mL of AA (0.1 mM). Then, 0.5 mL of HAuCl_4_ (1 mM) was added through a syringe pump at 25 µL/min rate under stirring conditions. After adding the HAuCl_4_ solution, the reaction was stirred at room temperature for about 30 min until the UV–vis spectra of the solution remained unaltered. Next, the sample was centrifuged at 5000 g and washed with milli-Q water twice. Finally, the pellet was re-suspended in 1 mL of milli-Q water for further characterization. For the synthesis of hybrid AuAg nanotubes, the procedure was the same, except the amount of HAuCl_4_ (1 mM) was less.

Solutions containing the 1D AuAg nanostructures were ultrasonicated for about 15 min and deposited on 15 nm thick Si_3_N_4_ membrane grids for STEM-EELS investigations. A hydrogen plasma cleaning using a Plasma Etch^TM^ plasma cleaner was applied prior to the EELS analyses to eliminate the organic residues that were present from the synthesis procedure. It should be mentioned here that the applied ultrasonication procedure may cause breakage of the longest nanotubes.

### 2.2. EELS Acquisition and Data Processing

Electron energy-loss spectroscopy (EELS) in a scanning transmission electron microscope (STEM) that is equipped with a monochromator is an ideal technique for studying the plasmonic responses of nanostructures as this technique has access to high spatial and energy resolutions [41]. Probe-corrected FEI™ Titan 60–300 STEM equipment was used for the EELS analysis and operated at 80 kV. The microscope was equipped with a high-brightness X-FEG gun, a Wien filter monochromator, and a Gatan™ Tridiem 866 ERS energy filter. A collection angle of 32 mrad and a dispersion of 0.01 eV per channel were used during the acquisition. Typical energy resolutions (full width at half of the maximum of the ZLP) of the measurements were better than 150 meV. EEL spectra were acquired using the spectrum imaging (SI) method [42,43] in which a sub-nanometer electron probe was scanned over the area of interest with a constant displacement of 4–6 nm.

EELS data was processed by a used a spectral unmixing (SU)-based routine of vertex component analysis (VCA) [31,44,45,46], which was implemented in the HyperSpy [47] multi-dimensional data analysis toolbox.

### 2.3. Simulations

Throughout the paper, we used boundary element method (BEM) [48,49] simulations to understand the effects of shape, composition, and environment (substrate) on the plasmonic properties of hollow 1D AuAg nanostructures. All BEM simulations were done using the MNPBEM Matlab^TM^ toolbox, which was developed by Hohenester [50]. The optical constants of the bulk metals were taken from Johnson & Christy [51] and modified according to Ref. [52] for the AuAg alloys. The size effects on the dielectric properties were also taken into account during the BEM simulations and assumed an increase in the damping constant in the Drude model with a reduction in the particle size due to the electron confinement effects [53].

## 3. Results and Discussion

**Figure 1:**
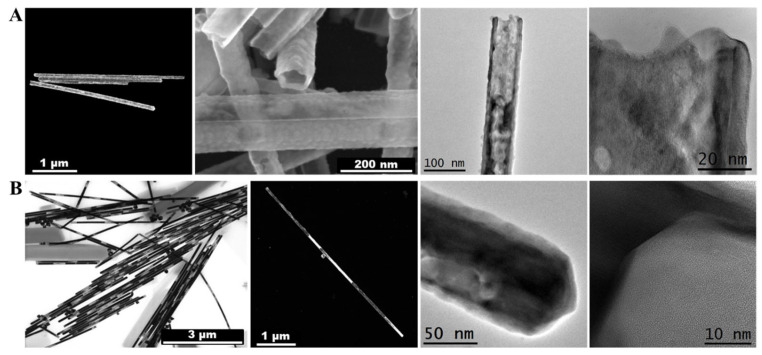
Microstructure of AuAg nanotubes. (**A**) HAADF-STEM, SEM, TEM, and HRTEM micrographs of the hollow nanotubes, respectively. (**B**) BF-STEM, HAADF-STEM, TEM, and HRTEM micrographs of the hybrid nanotubes, respectively.

The general microstructural features of the AuAg-prepared nanotubes are presented in Figure 1. The upper row (Figure 1A) shows the high-angle annular dark field (HAADF) STEM, SEM, TEM, and high-resolution TEM (HRTEM) micrographs that were obtained from the completely hollow AuAg nanotubes. Interestingly, the images reveal that (i) the nanotubes with lengths up to several micrometers that preserved the penta-twinned structure of the Ag nanowires were used as sacrificial templates and (ii) highly crystalline nanotubes with a wall thickness of ~10 nm and occasional pores along the walls. STEM-EDX point analyses obtained from the different parts of the hollow nanotubes revealed that completely hollow AuAg nanotubes have a chemical composition of about 60 ± 8 at.% Au and ~40 ± 8 at.% Ag. Bright-field (BF) STEM, HAADF-STEM, TEM, and HRTEM micrographs obtained from the hybrid AuAg nanotubes are shown in the lower row (Figure 1B), which clearly reveals that these nanotubes constitute a sequential formation of hollow parts within the solid Ag nanowire templates and solid parts. Hollow parts are composed of AuAg external walls formed after the galvanic replacement of Ag with Au, and the solid parts are pure Ag covered by an AuAg thin shell (see the STEM energy dispersive X-ray spectroscopy (EDX) results that are presented in Figures S1 and S2). It is also revealed in Figure 1B that hybrid AuAg nanotubes preserve the penta-twinned structure and are highly crystalline. As mentioned in the experimental procedure section, these 1D structures are synthesized by following the procedure reported in [38]. Very recently, Canepa et al. [54] reported anisotropic galvanic replacement reactions in Ag nanowires, synthesizing similar hybrid nanotubes as the present study. Here, we present a detailed investigation about the nanoscale distribution of plasmon resonances and plasmon mode interactions of such 1D nanostructures.

**Figure 2:**
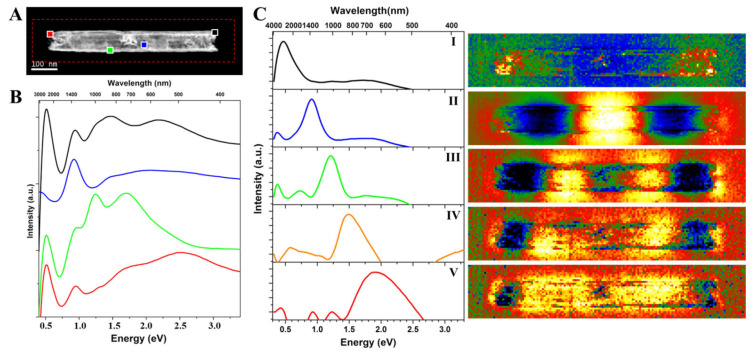
Plasmonic properties of a completely hollow AuAg nanotube. (**A**) HAADF-STEM micrograph of the AuAg nanotube which is 84 nm in diameter and 665 nm in length. The area of the EELS SI is indicated with a red rectangle. (**B**) Background subtracted selected area EEL spectra of different locations marked in (**A**). (**C**) Spectra and corresponding abundance maps of five plasmonic components obtained by VCA processing.

As seen in the above-presented micrographs, both completely hollow and hybrid AuAg nanotubes have lengths of several microns, which is quite impractical for nanoscale EELS mapping due to the long acquisition time and stability (drift) issues. Therefore, we tried to choose shorter nanotubes yet also keep the general features. Figure 2 shows the plasmonic properties of a completely hollow AuAg nanotube which is 84 nm in diameter (wall thickness: ~10 nm) and 665 nm in length (Figure 2A). Spatially resolved plasmonic properties of the nanotube were studied by obtaining an EELS spectral imaging (SI) over the area indicated with a red rectangle in Figure 2A. Figure 2B shows the background-subtracted selected area EEL spectra of different locations depicted in Figure 2A, revealing various plasmon peaks at ~0.5 eV, ~0.9 eV, ~1.2 eV, ~1.45 eV, ~1.7 eV, ~2.2 eV, and ~2.54 eV. It should be stressed here that one needs to take extra precautions while applying a background subtraction routine by using the power law [55] since the energy of the plasmon peaks are close to the zero-loss peak.

Obtained EELS data were processed by using a spectral un-mixing routine based on the vertex component analysis (VCA) algorithm. Figure 2C shows spectra of five different plasmon components and their corresponding abundance maps obtained by applying VCA analysis to the EELS data of the completely hollow AuAg nanotube. The plasmon components obtained by the VCA reveal the presence of Fabry–Perot resonator resonances [56] and LSPR for the AuAg nanotube (note that the colors of the spectra in Figure 2C do not represent the colored regions marked in Figure 2A). Fabry–Perot resonator modes in such quasi-1D metallic structures (or nanoantennas) consist of the propagation as well as the reflection of plasmons [56]. First-, second-, and third-order modes (components I, II, and III) are located at ~0.5 eV, ~0.9 eV, and ~1.2 eV, respectively. It should be emphasized here that the Fabry–Perot resonances seem to be most intense inside the nanotube, which is clearly revealed in the abundance maps of components II (second-order mode) and III (third-order mode). By looking at the abundance map of component IV, which is located at ~1.5 eV, this mode is more like a fourth-order mode than a LSPR mode. A wide peak located between 1.4 eV and 2.7 eV with a maximum at ~1.9 eV (component V) is associated with an LSPR mode, and its corresponding abundance map shows its distribution throughout the nanotube. Such plasmon distribution maps with a nanoscale resolution are obtained for the first time for metal. The findings reported here are quite similar to our previous report on AuAg nanoboxes [31], where the distribution of highly intense plasmon resonances in and around the nanostructures can be clearly observed, and their plasmonic properties are enhanced because of an increased intensity and a further reach. As mentioned earlier, the analyzed nanostructure is shorter than most of the nanotubes shown in Figure 1A, which was selected due to experimental convenience. One may suggest that analyzing a longer hollow nanotube would reveal a higher number of plasmonic Fabry–Perot resonator modes but also reveal quite similar general plasmonic features. Likewise, a shorter hollow nanotube would reveal the presence of a lower number of Fabry–Perot resonator modes. The fact that hollow 1D nanostructures have highly intense Fabry–Perot and LSPR modes at the inner and outer parts of the nanotube suggests that they can be good alternatives in different applications of plasmonic nanoantennas [11,12,14,17].

We continued with the simulations of plasmonic properties in 1D metal nanostructures. We did not conduct EELS experiments on Ag nanowires as their properties are widely studied in the literature; however, we simulated the plasmonic properties of an Ag nanowire that is the same size as the hollow AuAg nanotube as a reference. By using this reference, we also studied the differences between the solid and hollow Ag nanostructures and the effects of the substrate (i.e., 15 nm thick SiN_x_ TEM grids) presence, all of which are discussed in detail in the Supplementary Materials section (Figures S3–S5).

**Figure 3:**
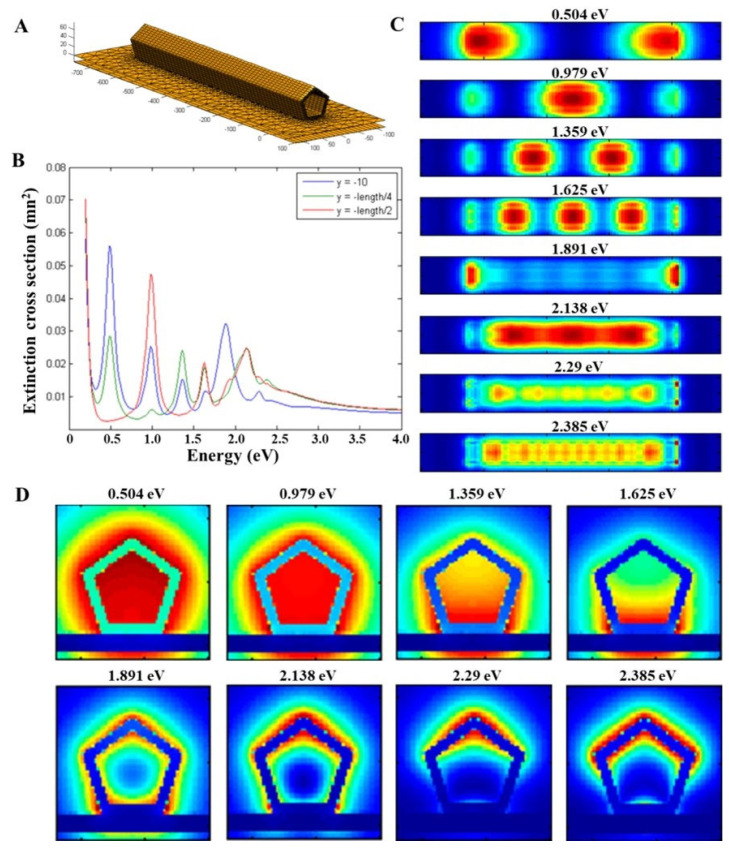
BEM simulations of the completely hollow AuAg nanotube. (**A**) Structural model of the BEM-simulated AuAg nanotube with a length of 665 nm, a diameter of 84 nm, and 10 nm thick walls standing on a 15 nm thick Si_3_N_4_ substrate. (**B**) Simulated local EEL spectra obtained at the tip (in blue), at a quarter of the length (at ~166 nm, in green), and at the center (at 332.5 nm, in red) of the AuAg nanotube. (**C**) BEM-simulated plasmon maps of eight different modes located at 0.504 eV, 0.979 eV, 1.359 eV, 1.625 eV, 1.891 eV, 2.139 eV, 2.29 eV, and 2.385 eV from the in-plane view. (**D**) BEM-simulated plasmon maps of the same modes from the cross-sectional view.

In Figure 3, BEM simulation results of the AuAg nanotube with a composition of 60 at.% Au and 40 at.% Ag (in accordance with the chemical analyses done by STEM-EDX) with 10 nm thick continuous walls are presented. The simulated AuAg nanotube has the same dimensions as the experimentally studied one (Figure 2). Several assumptions had to be made during the simulations: (i) the distribution of Au and Ag throughout the nanotube is considered homogeneous and (ii) we discarded the possibility of pores around the walls. Figure 3A shows the structural model of the AuAg nanotube standing on a 15 nm thick Si_3_N_4_ substrate. Figure 3B shows the BEM-simulated EELS spectra obtained at the tip (near the edge, in blue) at one-quarter of the nanotube length (at ~166 nm, in green) and the center (at 332.5 nm, in red), which reveals the presence of several peaks. It is worth noting that there are almost no visible peak at energies higher than 2.5 eV, unlike in the EEL spectra obtained from pure Ag (Figures S4–S6). The plasmon peaks at high energies are diminished due to a mechanism called plasmon damping [57], in which the overlapping of the onset of the interband transitions with the LSPRs of Au causes a decrease in LSPR intensity at higher energies [57,58]. Another feature to note is that the intensity of the EELS signal below 0.5 eV increases toward lower energy. This might suggest the possible presence of a dark, plasmonic breathing-like mode at this energy range, yet it is hard to tell by just looking at these simulated EEL spectra. BEM-simulated plasmon maps of eight different modes are presented for the AuAg nanotube (Figure 3C). Fabry–Perot resonator modes up to fourth order are revealed, which shows that these modes become most excited inside the nanotube. These maps obtained by BEM simulations are quite similar to the experimentally observed abundance maps of VCA (Figure 2C), where it is shown that the Fabry–Perot modes are highly intense inside the nanotube. The LSPR mode located at 1.891 eV is confined to the tips of the nanotube, and the LSPR mode located at 2.138 eV seems to be present all over the AuAg nanotube. Moreover, plasmon maps of two other LSPRs of different polar modes located at 2.29 eV and 2.385 eV are shown in this figure. As discussed in the Supplementary Materials part and reported in the literature [59,60,61], the presence of a substrate splits the LSPR modes into proximal and distal modes. Figure 3D shows the BEM-simulated plasmon maps of the AuAg nanotube obtained by the beam incident on the pentagonal cross-section to better understand the proximal and distal modes. As seen in this figure, cross-sectional plasmon maps of the eight different modes (whose planar views are presented in Figure 3C) are shown, where the 15 nm thick substrate is clearly visible. The first- and second-order Fabry–Perot modes have a homogeneous distribution throughout, both at the inner and outer parts of the AuAg nanotube. However, third- and fourth-order Fabry–Perot modes have a highly intense distribution that is confined to the lower parts (which are in contact with the substrate) of the nanotube, resembling proximal modes. The first LSPR mode located at 1.891 eV is a proximal mode with a somewhat homogeneous distribution of plasmon resonances along the inner and outer parts of the nanotube wall. The other three LSPR modes located at 2.138 eV, 2.29 eV, and 2.385 eV can be clearly identified as distal modes from these cross-sectional plasmon maps. The mode located at 2.29 eV is confined to the distal corner with some contributions from the upper edges, whereas the mode located at 2.385 eV is more like an edge LSPR mode generated from the distal edges. BEM-simulated 3D maps of these modes are presented in the Figure S6.

**Figure 4:**
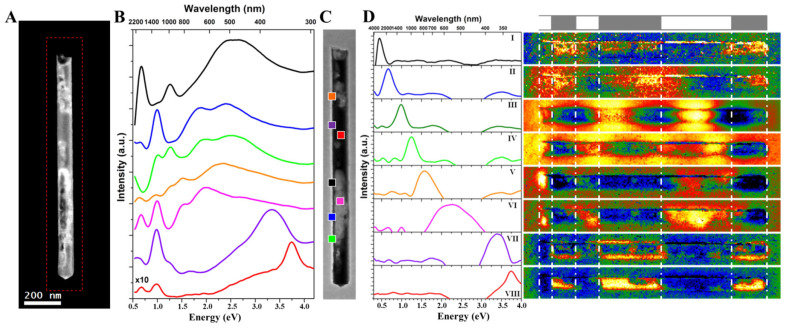
Plasmonic properties of a hybrid AuAg nanotube. (**A**) HAADF-STEM micrograph of the hybrid AuAg nanotube which is 89 nm in diameter and 1.24 µm in length. The area of the EELS SI is indicated with a red rectangle. (**B**) Background subtracted selected area EEL spectra of different locations marked in (**C**), which is the EELS SI taken from the red rectangle in (**A**). (**D**) The spectra and corresponding abundance maps of eight plasmonic components obtained by VCA processing. Hollow and solid sections are marked with white dashed lines.

Figure 4A shows a 1.24 µm long hybrid AuAg nanotube with a diameter of 89 nm, where EELS SI is obtained over the area indicated with a red rectangle. Figure 4B shows the background-subtracted selected area EEL spectra of different locations indicated in Figure 4C, which is the EELS SI obtained from the region marked with a red rectangle in Figure 4A. As seen in the local EEL spectra, this hybrid AuAg nanotube contains multiple plasmon resonances with energies ranging from ~0.6 eV to 3.8 eV. Unlike the above presented completely hollow nanotube, the hybrid nanotube contains plasmon resonances that are generated with higher energies up to the bulk plasmon resonance of Ag located at ~3.8 eV (shown in red). It is also worth noting that the EEL spectrum obtained from the area indicated with a purple square reveals the presence of a LSPR mode of Ag located at ~3.36 eV. By comparing the EEL spectra obtained from the hollow and solid parts of the hybrid nanotube (shown in pink and red, respectively), one can see that the plasmon resonances of these regions are quite distinctive from one another.

Figure 4D shows the eight different plasmon components and their corresponding abundance maps obtained by applying VCA analysis to the EELS data of the hybrid AuAg nanotube. It should also be noted here that the colors of the spectra that are obtained via VCA in Figure 4D do not represent the colored regions in Figure 4C. The first thing to highlight in this figure is that the EEL spectrum and plasmon map of the first-order component is located at 0.44 eV and could not be observed after the zero-loss peak subtraction by Power Law. The components obtained by VCA reveal the second-, third- and fourth-order resonator modes (components II, III, and IV). As shown experimentally and by BEM simulations for the completely hollow AuAg nanotubes, the Fabry–Perot resonator modes are generated intensely inside the hollow nanotubes. The abundance map of the third-order mode (component III) clearly confirms such behavior for the hybrid AuAg nanotube, where the same mode has a much higher intensity at the hollow part (on the right) compared with the solid part (on the left) along the same nanotube. Intriguingly, the distribution of resonator-like modes is not symmetrical for the hybrid AuAg nanotube due to the plasmon hybridization between the hollow and solid parts of this 1D nanostructure. Yazdi et al. [36] reported such a plasmon hybridization in partially hollow, shorter AgAu nanorods. Here, we show that symmetry breaking in resonant modes can be manifested clearly for higher-order modes in longer and consequential hybrid 1D nanostructures. Liang et al. [62] also reported a similar asymmetric plasmon resonance distribution in asymmetric silver “nanocarrot” structures. Aside from these studies, plasmon mode coupling of higher-order modes was also observed in dimer/complex structures [30,63,64,65,66]. For instance, Schubert et al. [63] obtained a symmetry-break in AuAg nanowire dimers separated by 10 to 30 nm and reported a surface plasmon coupling between the second-order mode and third-order mode of two individual nanowires present in the asymmetric dimer, resulting in a bonding–antibonding mode pair. A component located at ~2.3 eV that covers a wide range of energies between 1.6 and 3.1 eV is obtained (component VI) by VCA, and its corresponding abundance map reveals that this component is associated with the LSPR mode of the hollow parts. Components related to the surface and bulk plasmon resonances of Ag are presented in components VII and VIII, respectively, along with their distribution, indicating that they are only present in or on the surface of the solid parts.

**Figure 5:**
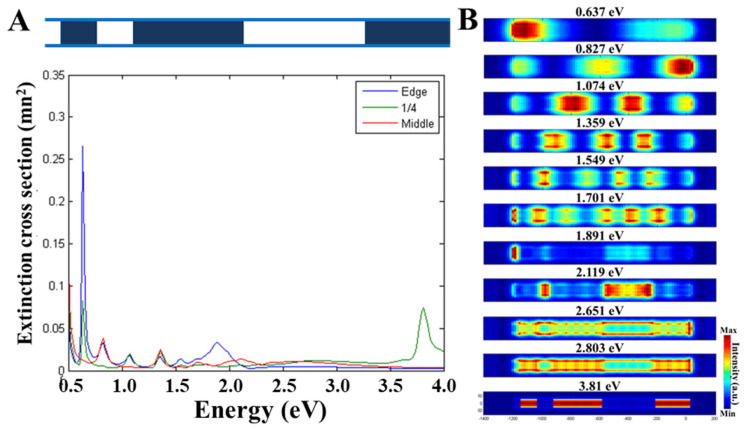
BEM simulations of the hybrid AuAg nanotube. (**A**) Schematic drawing of the BEM-simulated AuAg nanotube with a length of 1240 nm, a diameter of 89 nm, and 10 nm thick walls, along with the simulated local EEL spectra obtained at the tip (in blue), at a quarter of the length (at ~310 nm, in green), and at the center (at 620 nm, in red) of the AuAg nanotube. (**B**) BEM-simulated plasmon maps of 11 different modes located at 0.637 eV, 0.827 eV, 1.074 eV, 1.359 eV, 1.549 eV, 1.701 eV, 1.891 eV, 2.119 eV, 2.651 eV, 2.803 eV, and 3.81 eV from the in-plane view.

Figure 5 shows the BEM simulation results obtained from the hybrid AuAg nanotube presented in Figure 4. During the BEM simulations, we used an AuAg nanotube with a length of 1240 nm, a diameter of 89 nm, and 10 nm thick walls (the same sizes as the experimentally investigated nanotube) with well-defined porous and hollow parts, unlike the experimentally investigated one. The simulated hybrid AuAg nanotube model has a sequence of 50 nm hollow, 110 nm solid, 110 nm hollow, 340 nm solid, 375 nm hollow, and 245 nm solid parts, where the hollow parts are composed of 60% Au and 40% Ag, and the solid parts are pure Ag. Figure 5A shows the simulated local EEL spectra obtained at the tip (in blue), at a quarter of the length (at ~310 nm, in green), and at the center (at 620 nm, in red) of the AuAg nanotube where numerous plasmon peaks are observed.

By taking these peaks into account, we have obtained BEM-simulated maps of 11 different plasmon modes (Figure 5B) from the in-plane view. The first six modes have plasmon resonances between 0.637 eV and 1.701 eV and are Fabry–Perot resonator modes. It should be noted that the experimental results presented in Figure 4 reveal the presence of only four different resonator modes. Even though the number of the plasmon modes is not the same, the distribution of these plasmon resonances is quite similar for the experimental and BEM-simulated results. BEM-simulated results confirm symmetry breaking for the Fabry–Perot resonator modes due to the plasmon hybridization between the hollow and solid parts, and the experimentally observed plasmon intensely differs within the hybrid AuAg nanotube. A LSPR mode located at 1.891 eV is found to be mostly confined to the hollow tip of the hybrid nanotube, the distribution of which is quite similar to the experimentally observed plasmon mode located at ~1.6 eV. Hollow parts of the simulated nanotube have a LSPR mode located at 2.119 eV. As seen in the BEM-simulated plasmon maps presented in Figure 5B, two other LSPR modes generated from the Ag-rich regions are located at 2.651 eV and 2.803 eV, and the bulk plasmon resonance for the Ag parts are located at 3.81 eV. Due to the consistency between the experimental and simulation results, one can thoroughly understand the detailed optical properties of different photonic systems [67].

With the local plasmonic properties of these hybrid nanotubes and the recent advancement in the fabrication of hollow nanostructures via nanosecond laser [68] or localized electron beam irradiation, a new era of the controlled tailoring of codified plasmonic nanostructures with symmetric/asymmetric plasmonic properties can be initiated.

## 4. Conclusions

This paper reports the plasmonic properties and asymmetrical plasmon distributions in the 1D hollow nanostructures of two different AuAg nanotubes, including completely hollow nanotubes and hybrid solid/hollow coded nanotubes comprising the sequential formation of solid Ag parts and hollow AuAg parts.

The completely hollow AuAg nanotube revealed the presence of several Fabry–Perot resonator modes and LSPR modes where the Fabry–Perot modes were very intense inside the nanotube. The presence of multiple Fabry–Perot modes and LSPR modes are observed on a hybrid solid/hollow coded AuAg nanotube. The LSPRs in the hybrid AuAg nanotube have been generated distinctively from the hollow and solid parts of the nanotube, which creates a way to control a broad range of plasmon resonances with one single nanostructure. The periodicity of the Fabry–Perot modes is disrupted in this hybrid AuAg nanotube due to plasmon hybridization by the interaction of the solid and hollow parts, which resulted in an asymmetrical plasmon distribution in a single 1D nanostructure.

We believe that understanding the plasmon resonances of such nanostructures and the possibility of codifying the presence of hollow cavities in the nanotubes by applying laser or electron beam irradiation in localized areas opens a new field in plasmonics for the accurate and at-will control of plasmon resonances.

## Data Availability

The data presented in this study are openly available in ZENODO at https://doi.org/10.5281/zenodo.7699893 (accessed on 7 February 2023).

## Supplementary Materials

The following supporting information can be downloaded at: https://www.mdpi.com/article/10.3390/nano13060992/s1. STEM-EDX analyses of the hybrid nanotubes. BEM simulations of pure Ag nanowires and Ag nanotubes in vacuum. BEM simulations of Ag nanotubes on a 15 nm thick Si_3_N_4_ substrate. The following file is available free of charge: Supporting Info_Hybrid NTs Plasmonics (PDF). References [32,36,59,61] are cited in the Supplementary Materials.
