# Supplementary material for: Asymmetrical Plasmon Distribution in Hybrid AuAg Hollow/Solid Coded Nanotubes
Source: Nanomaterials (Basel). 2023 Mar 9;13(6):992. doi: 10.3390/nano13060992 (PMC10051431; doi:10.3390/nano13060992)
Supplement: Supplementary file 1 [file nanomaterials-13-00992-s001.zip › nanomaterials-2236466-supplementary.pdf]

Supplementary Materials

Asymmetrical Plasmon Distribution in Hybrid AuAg Hollow/Solid Coded Nanotubes

Aziz Genç [1], Javier Patarroyo [1], Jordi Sancho-Parramon [2], Raul Arenal [3,4,5], Neus G. Bastús [1], Victor Puntes [1,6,7] and Jordi Arbiol [1,7,*]

[1] Catalan Institute of Nanoscience and Nanotechnology (ICN2), CSIC and BIST, Campus Universitat Autònoma de Barcelona, 08193 Barcelona, Spain
[2] Rudjer Boskovic Institute, 10000 Zagreb, Croatia
[3] Instituto de Nanociencia y Materiales de Aragon (INMA), CSIC-U de Zaragoza, 50009 Zaragoza, Spain
[4] Laboratorio de Microscopias Avanzadas (LMA), Universidad de Zaragoza, 50018 Zaragoza, Spain
[5] ARAID Foundation, 50018 Zaragoza, Spain
[6] Vall d'Hebron Institut de Recerca (VHIR), 08035 Barcelona, Spain
[7] ICREA, 08010 Barcelona, Spain
* Correspondence: arbiol@icrea.cat

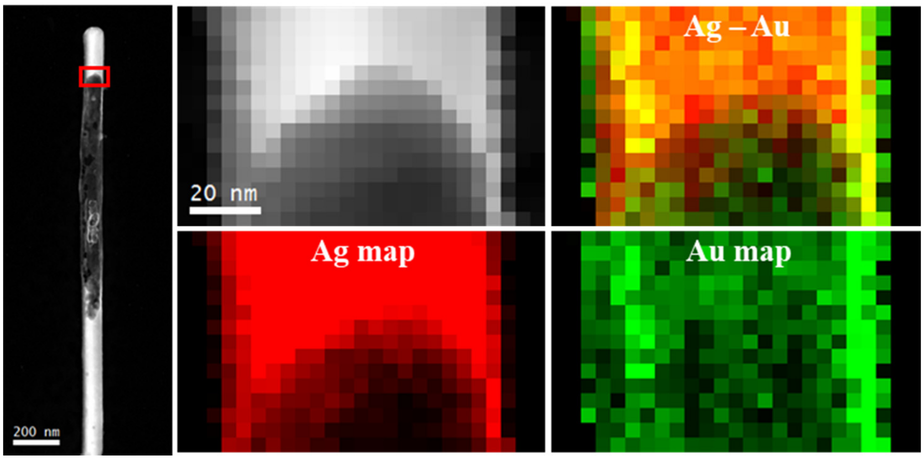

Figure S1. HAADF – STEM image of an individual hybrid AuAg nanotube and EDX elemental maps of Ag and Au over the area indicated with a red rectangle, along with their composite image.

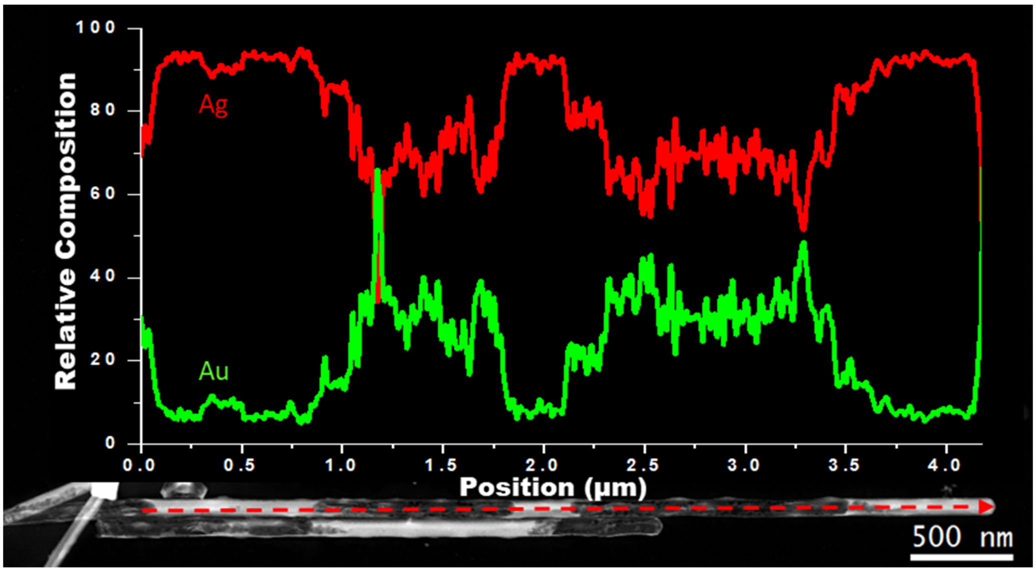

Figure S2. HAADF – STEM image of an individual hybrid AuAg nanotube and relative compositions of Ag (in red) and Au (in green) obtained by an EDX line scan along the red arrow.

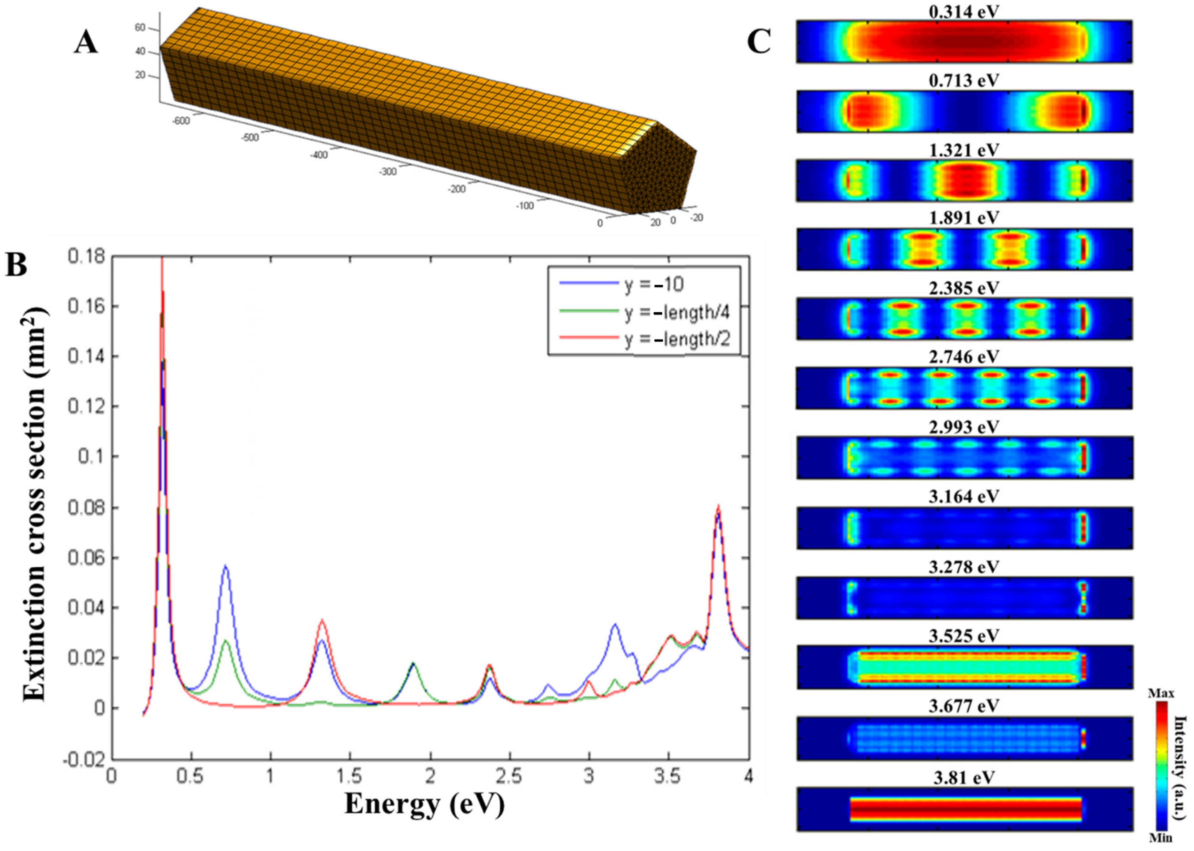

Figure S3. BEM simulations of Ag nanowire. A. Structural model of the BEM simulated Ag nanowire with a length of 665 nm and a diameter of 84 nm. B. Simulated local EEL spectra obtained at the tip, at a quarter of the length (at ~166 nm) and at the center (at 332.5 nm) of the Ag nanowire. C. BEM simulated plasmon maps of 12 different plasmon modes for the Ag nanowire. Note that the nanowire is standing in vacuum.

Since plasmon mapping of Ag nanowires/nanorods via EELS is studied extensively in the literature, we only conduct BEM simulations for Ag nanostructures. We simulate a Ag nanowire with the same dimensions as the completely hollow AuAg nanotube. Thanks to the applied BEM simulations, we also report the effects of hollow morphology for nanostructures standing in vacuum as well as the effects of presence of a silicon nitride substrate on the plasmonic properties of 1D Ag nanostructures.

Fig. S3A shows the structural model of the Ag nanowire that is used for the BEM simulations. The simulated nanowire has a pentagonal morphology with the same sizes of a 665 nm length and 84 nm diameter as the experimentally investigated (presented in Fig. 2 of the main

text), completely hollow AuAg nanotube. It should be noted here that the Ag nanowire is standing in vacuum. Fig. S3B shows the BEM simulated EELS spectra obtained at the tip (near the edge, in blue), at the one quarter of the nanowire length (at ~166 nm, in green) and at the center (at 332.5 nm, in red) of the nanowire. As seen in these EEL spectra, presence of several peaks are observed at the different locations of the Ag nanowire. At the first instance, the presence of at least 12 peaks can be observed. BEM simulated plasmon distribution maps of these peaks are presented in Fig. S3C. The lowest energy peak is located at 0.314 eV and it is present in all three locations, suggesting a presence of a dark plasmonic breathing mode for this simulated Ag nanowires. Second peak located at 0.713 eV is present at the tip with high intensity. The periodicity of the peak locations for some of the peaks listed above suggests the presence of Fabry-Perot resonator type modes, which is quite expected for an Ag nanowire. And, by looking at the distribution of the plasmon resonance peak located at 0.713 eV, it can be suggested that this is the first order resonator mode. A third peak located at 1.321 eV is present at the tip and at the center of the nanowire and is identified as the second order resonator mode. Plasmon distribution maps of the resonator modes up to sixth order are present in Fig. S3C (located at ~1.89 eV, ~2.38 eV, ~2.74 eV and ~2.99 eV, respectively), with some other localized surface plasmon resonance modes (located at ~3.16 eV, ~3.28 eV, ~3.52 eV and ~3.68 eV) and bulk plasmon mode of Ag at 3.81 eV.

BEM simulation results of an Ag nanotube are presented in Fig. S4, allowing us to understand the effects of hollow morphology on the plasmonic properties. Fig. S4A shows the structural model of the Ag nanotube that is used for the BEM simulations. The simulated nanotube also has a pentagonal morphology with the same sizes of a 665 nm length and 84 nm diameter with a 10 nm thick continuous wall. It is standing in vacuum. Fig. S4B shows the BEM simulated EELS spectra obtained at the tip (near the edge, in blue), at the one quarter of the nanotube length (at ~166 nm, in green) and at the center (at 332.5 nm, in red) of the nanotube. At the first instance, one can see that the breathing plasmon mode observed for the Ag nanowire is not present at the BEM simulated EEL spectra of the Ag nanotube. Moreover, it is clear that plasmon resonances shifted to lower energies compared to solid Ag nanowire due to plasmon hybridization between solid and cavity modes [1]. As seen in these EEL spectra, presence of several peaks are observed at the different locations of the Ag nanotube. It should be pointed out here that we did not take the instrumental broadening into account during these BEM

simulations. Some of these peaks with very similar energy values would merge together in the experimental EELS measurements.

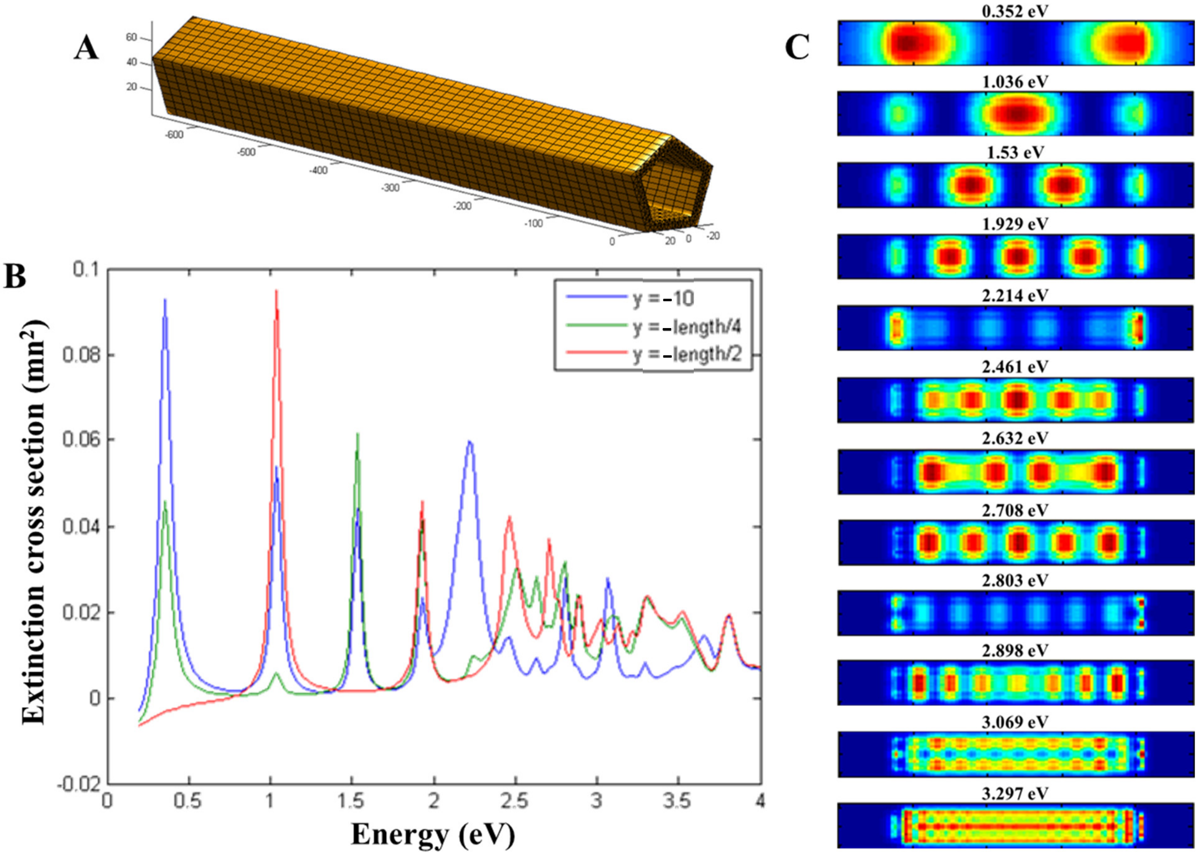

Figure S4. BEM simulations of Ag nanotube. A. Structural model of the BEM simulated Ag nanotube with a length of 665 nm and a diameter of 84 nm. B. Simulated local EEL spectra obtained at the tip, at a quarter of the length (at ~ 166 nm) and at the center (at 332.5 nm) of the Ag nanotube. Note that the nanotube is standing in vacuum. C. BEM simulated plasmon maps of 12 different plasmon modes for the Ag nanotube.

BEM simulated plasmon maps of 12 different peaks located at 0.352 eV, 1.036 eV, 1.53 eV, 1.929 eV, 2.214 eV, 2.461 eV, 2.632 eV, 2.708 eV, 2.803 eV, 2.898 eV, 3.069 eV and 3.297 eV in Fig. S4B are shown in Fig. S4C. The mode located at 0.352 eV is the first order Fabry-Perot type resonator mode, which was located at 0.713 eV for the solid Ag nanowire. Such a shift reveal the effects of the plasmon hybridization in hollow nanostructures [2]. The distributions of second, third and fourth order Fabry-Perot modes

located at 1.036 eV, 1.53 eV and 1.929 eV, respectively, are clearly seen in this BEM simulated plasmon maps. It should be noted here that these Fabry-Perot resonator modes are most efficiently excited at the inside of the nanotube. The LSPR mode located at 3.164 eV in the solid Ag nanowire shifted significantly to 2.214 eV in the hollow Ag nanotube, which is highly confined at the tips of the nanotube. The nature of the modes located at 2.461 eV, 2.708 eV and 2.898 eV is not clear in these plasmon maps, i.e. they are most probably high order Fabry-Perot modes, yet they may be LSPR modes as well. In any case, it is clear that they have intense resonances both at the inner and outer parts of the nanotube. Similarly, a LSPR mode located at 2.632 eV have high intensities at the inner and outer parts of the nanotube. Another LSPR mode located at 2.803 eV is mostly confined at the tips with some contributions from the other parts of the nanotube. The plasmon maps of the modes located at 3.069 eV and 3.297 eV reveals that these modes have multipolar contributions.

After revealing the differences between a solid and hollow 1D nanostructure standing in vacuum, we continue with the addition of a substrate in order to discuss its effects on the plasmonic properties of hollow 1D nanostructures. As reported in the literature, plasmon modes of the nanostructures interact with the sample resulting in the formation of distal and proximal modes [3, 4]. Fig. S5A shows the structural model of the Ag nanotube (655 nm long, 84 nm wide with 10 nm thick walls) standing on a 15 nm thick Si_3N_4 substrate, which is used during BEM simulations of the Ag nanotubes. Fig. S5B shows the BEM simulated EELS spectra obtained at the tip (near the edge, in blue), at the one quarter of the nanotube length (at ~166 nm, in green) and at the center (at 332.5 nm, in red) of the nanotube. It is seen that all the peaks shifted to lower energies except the lowest energy peak corresponding to the first order Fabry-Perot resonator mode located at ~0.52 eV, which is located at 0.352 eV for the Ag nanotube standing in vacuum. As there is no study about the plasmonic properties of hollow 1D nanostructures, we are not sure whether this is caused by a numerical accuracy or it is typical for such nanostructures. The presence of many different peaks obtained at different parts of the Ag nanotube standing on a Si_3N_4 substrate is shown in this figure. Since there are more than 10 peaks located between 2 eV and 3.8 eV, we do not mention all the peaks with their energy values but one can easily distinguish

several sharp peaks located at ~0.52 eV, ~1.0 eV, ~1.4 eV, ~1.7 eV eV, ~2.0 eV and ~2.4 eV among many others.

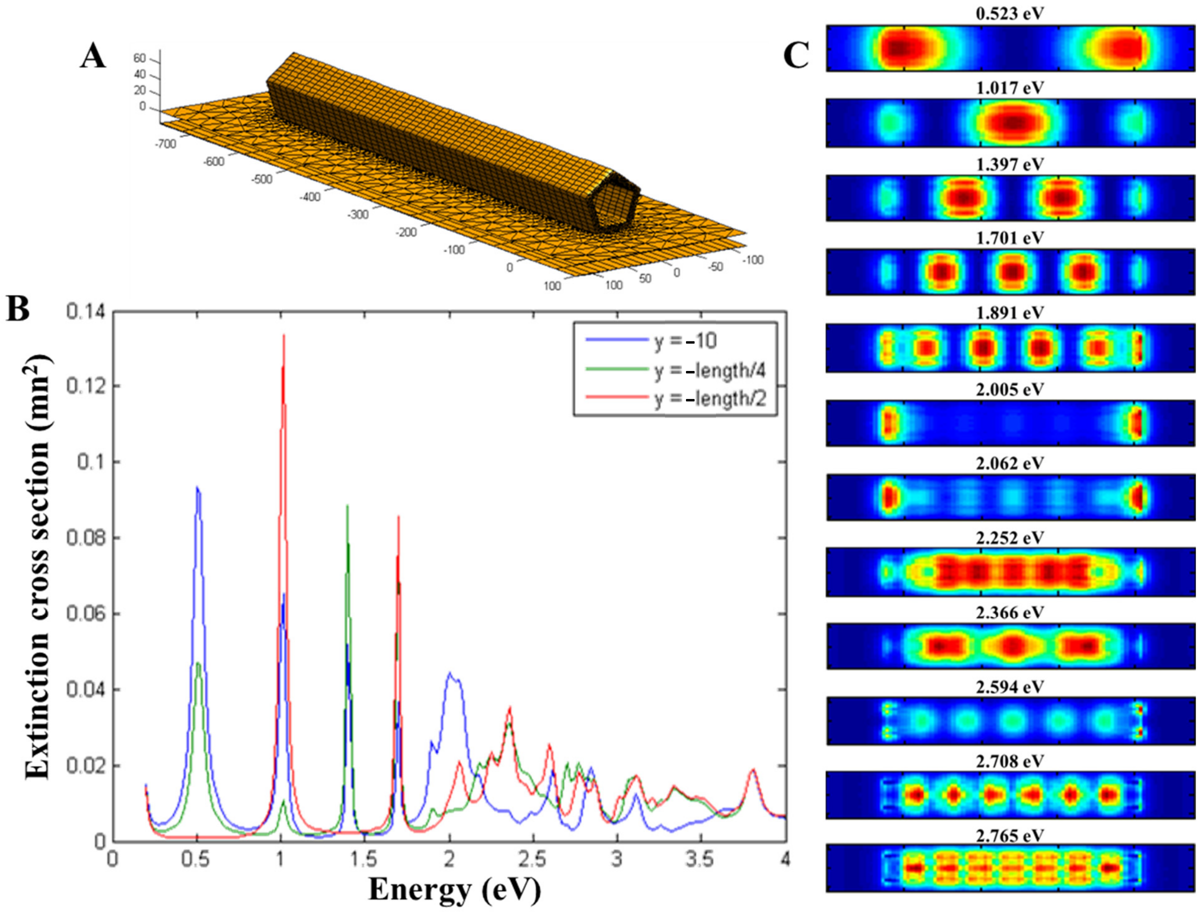

Figure S5. BEM simulations of an Ag nanotube on 15 nm thick Si_3N_4 substrate. A. Structural model of the BEM simulated Ag nanotube with a length of 665 nm and a diameter of 84 nm, with 10 nm thick walls, standing on a 15 nm thick Si_3N_4 substrate. B. Simulated local EEL spectra obtained at the tip, at a quarter of the length (at ~166 nm) and at the center (at 332.5 nm) of the Ag nanotube. C. BEM simulated plasmon maps of 12 different plasmon modes for an Ag nanotube standing of a Si_3N_4 substrate.

BEM simulated plasmon maps of 12 different peaks located at 0.523 eV, 1.017 eV, 1.397 eV, 1.701 eV, 1.891 eV, 2.005 eV, 2.062 eV, 2.252 eV, 2.366 eV, 2.594 eV, 2.708 eV and 2.765 eV in Fig. S5B are shown in Fig. S5C. As mentioned before, the peaks shifted

to lower energies in general except the one located at 0.523 eV. Its BEM simulated map shows that this is the first order Fabry-Perot resonator mode and it is mostly excited inside the nanotube. Second, third, fourth and fifth order Fabry-Perot modes, which are located at 1.017 eV, 1.397 eV, 1.701 eV and 1.891 eV, respectively, reveals the similar distribution of plasmon resonances, i.e. they are mostly intense at the inner part of the Ag nanotube along with intense presence at the outer parts. Two LSPR modes that are located at ~2 eV and 2.062 eV are highly confined at the tips of the nanotube. As mentioned before, after taking the instrumental broadening into account, which is about 130 meV for the experimentally obtained EELS maps, these two peaks with an energy difference of 57 meV would merge together. BEM simulated plasmon maps of some other LSPR modes located at various energies are shown in this figure. Similar to the discussions about BEM simulated plasmonic properties of cuboid AuAg nanostructures, we have used pure Ag as a playground in order to discuss the plasmonic property differences between solid and hollow 1D nanostructures and the effects of substrate presence on the plasmonic properties of the hollow 1D nanostructures. So far, we have shown that both solid (Ag nanowires) and hollow (Ag nanotubes) 1D nanostructures contain multiple Fabry-Perot type resonator modes and LSPR modes. The presence of a dark plasmonic breathing mode for the Ag nanowire is observed during the BEM simulations and we need to have a better understanding about this mode. In addition, we have observed that the energy of Fabry-Perot and LSPR modes shifted to lower energies for the hollow nanotube compared to those of the solid nanowires, due to plasmon hybridization between solid and cavity modes. It has been revealed that most of the plasmon modes (both Fabry-Perot type and LSPR) are excited most intensely from the inner parts of the hollow nanostructure. Implementation of a 15 nm thick Si3N4 substrate caused a furhter shift of the plasmon resonances of the Ag nanotubes to lower energies, except the first order Fabry-Perot mode which shifted to higher energies.

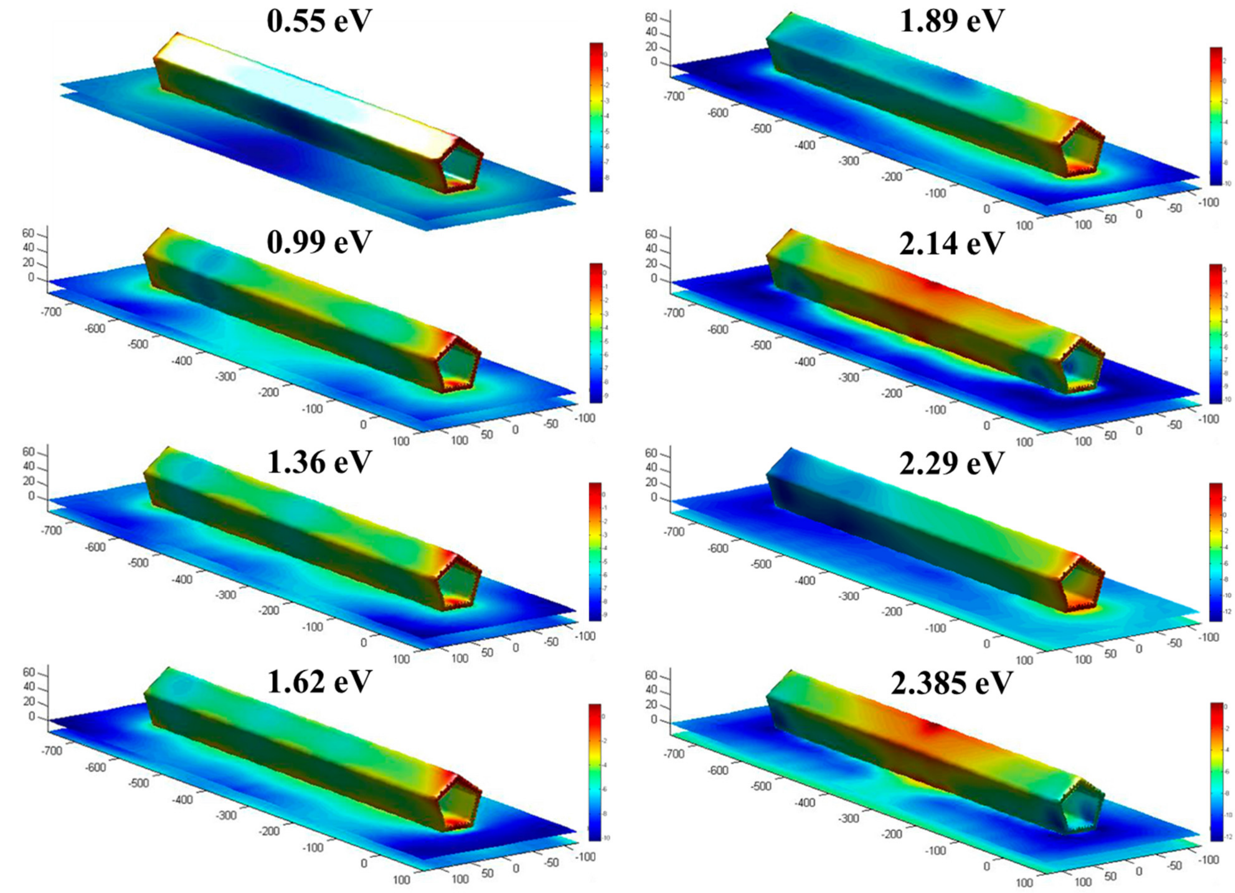

Figure S6. 3D BEM simulations of the hollow AuAg nanotube on 15 nm thick Si_3N_4 substrate.

References

[1] E. Prodan, C. Radloff, N. J. Halas, P. Nordlander. A hybridization model for the plasmon response of complex nanostructures. Science 302 (2003) 419-422.

[2] A. Genç, J. Patarroyo, J. Sancho-Parramon, R. Arenal, M. Duchamp, E.E. Gonzalez, L. Henrard, N.G. Bastús, R.E. Dunin-Borkowski, V.F. Puntes, J. Arbiol, J., Tuning the plasmonic response up: hollow cuboid metal nanostructures. ACS photonics, 3 (2016) 770-779.

[3] O. Nicoletti, F. de la Pena, R. K. Leary, D. J. Holland, C. Ducati, P. A. Midgley. Three-dimensional imaging of localized surface plasmon resonances of metal nanoparticles. Nature 502 (2013) 80 – 84.

[4] S. Zhang, K. Bao, N. J. Halas, H. Xu, P. Nordlander. Substrate-induced Fano resonances of a plasmonic nanocube: A route to increased-sensitivity localized surface plasmon resonance sensors revealed. Nano Letters 11 (2011) 1657-1663.
